# Does environmental attention differ during walking, jogging, and cycling in greenways? Evidence from eye movement responses to videos

**DOI:** 10.3389/fpsyg.2025.1665574

**Published:** 2025-10-09

**Authors:** Yidian Pan, Chang Liu, Yue Liu, Yuhan Liu, Jiayi Zhao, Yongrong Zheng, Songjun He

**Affiliations:** College of Landscape Architecture and Art, Fujian Agriculture and Forestry University, Fuzhou, Fujian, China

**Keywords:** greenway, physical activity, health promotion, visual attention, eye-movement tracking

## Abstract

**Introduction:**

Greenways accommodate various path-based physical activities (PPAs). This study aimed to examine the similarities and disparities in environmental attention and cognitive responses during different greenway PPAs.

**Method:**

The eye movements of 26 participants were recorded while watching a greenway video during walking, jogging, and cycling in counterbalanced sequences.

**Results:**

The results of the Kruskal–Wallis test indicate that (1) in terms of similarities, vegetation received the most attention with a higher number of fixations (rNF) and a greater total fixation duration (rTDF) compared to other areas of interest (AOIs). However, roads demanded more cognitive activities for spatial orientation as indicated by the longest average fixation duration (rAFD). (2) Regarding disparities, walkers showed a greater fascination with vegetation, displaying a higher rTDF than joggers and cyclists and a longer relative first fixation duration (rDFF) compared to other AOIs. Joggers demonstrated more concentrated attention with the lowest average blink rate (AB), producing a heatmap dominated by longer fixations, and they experienced a higher rAFD than walkers and cyclists on roads. Cyclists were more strongly attracted toward distant views, as evidenced by their flattened-shaped heatmap, and open water bodies drew their attention as their rNF on water exceeded that observed in other PPAs.

**Discussion:**

Therefore, clear and varied vegetation landscapes, distinct and noticeable roads, and open water surfaces offering distant views can better hold the attention of different PPA exercisers and minimize their mental fatigue. The research contributes to enhancing the role of greenways in promoting physical activity and fostering psychological wellbeing through activity-adapted environmental interactions.

## 1 Introduction

Greenways have been implemented in various countries and regions worldwide to promote physical activity and active transportation (East Coast Greenway, 2019; Blue Ridge Parkway, 2018; Department of Transport, and Tourism and Sport, 2018). Furthermore, survey data indicate that the introduction of greenways has a positive impact on the physical activity levels of nearby residents in most cases (Deng et al., 2023; Frank et al., 2019). However, existing studies primarily focus on overall physical activity levels, with insufficient attention given to the relationship between greenways and specific types of activities. As linear open spaces providing continuous pathways, greenways are particularly suitable for path-based physical activities (PPAs) such as walking, jogging, and cycling, which rely on accessible paths. Some studies have explored the relationship between greenway environmental features and specific activities like walking, jogging, and cycling. However, few studies compare these three activities within a unified framework. Although all three PPAs share similarities in terms of path dependence, they differ in exercise intensity and movement speed. Therefore, their environmental requirements theoretically share similarities and disparities. This knowledge gap impedes the comprehensive design and optimization of greenways aimed at encouraging diverse physical activities.

Current research relies heavily on objectively observed activity levels or preferences obtained through questionnaires, with limited exploration into exercisers’ environmental attention patterns during physical activity. However, the experience of exercise is significantly influenced by environmental attention. Attention is distributed both internally and externally during physical activity. Internally, attention is allocated to maintaining muscle tension, exercise intensity, and body balance; externally, it is directed toward the surrounding environment (Rogerson and Barton, 2015). A cluttered external environment may impose a higher cognitive load, reducing the available cognitive resources for sustaining motion and potentially impairing exercise performance. Conversely, an environment lacking stimuli may fail to effectively capture external attention, leading individuals to focus excessively on their physical state and increasing their rating of perceived exertion (RPE), which reflects greater mental fatigue related to sports (Han, 2020). Thus, appropriate environmental attention plays a crucial role in sustaining exercise performance and reducing psychological fatigue.

In this study, we developed an attention interpretation framework based on Attention Restoration Theory (ART) to clarify the different types of attention exhibited by exercisers during physical activity, the underlying cognitive mechanisms, and their relationships with eye-movement indicators. ART distinguishes between directed attention and undirected attention. Directed attention is a top-down cognitive process that requires volitional control, enabling individuals to maintain focus on specific tasks, such as navigation and spatial positioning, and is susceptible to causing mental fatigue. Undirected attention, in contrast, is a bottom-up, involuntary attentional mode typically elicited by inherently attractive environmental elements—such as water and vegetation. It does not require conscious effort and facilitates attention restoration and psychological relaxation. Currently, the interpretation of attention in eye-movement research remains ambiguous. The same eye-movement behavior can be interpreted differently depending on the context. For instance, prolonged fixation duration may indicate interest and exploration linked to undirected attention, or it may reflect increased cognitive load and task difficulty associated with directed attention. This ambiguity highlights the limitations of relying solely on eye-movement data for attentional inference; instead, a comprehensive interpretation must incorporate specific task demands and environmental contexts. In this paper, we adopt ART as a theoretical foundation for interpreting attention. Eye-movement patterns associated with undirected attention such as sustained fixations on natural elements are interpreted as indicators of interest driven by environmental attractiveness. Conversely, eye-movement features linked to directed attention, such as extended fixations on road-related cues and elevated blink rates are regarded as markers of cognitive load.

The observation of environmental attention is commonly conducted using eye-tracking technology, which effectively captures human visual behaviors such as fixations, saccades, and scanning patterns (Duchowski, 2003). Among various eye movement metrics, fixation is most reflective of environmental attention and is typically measured by duration and frequency. To evaluate the impact of different environmental elements on attention, specific areas of interest (AOIs) are defined according to the study’s objectives and subjects. By comparing the fixation metrics across different AOIs, the relative influences of the AOIs on attention can be determined. In research focused on urban green spaces (UGS), vegetation, water, roads, and buildings are often classified as AOIs. Additionally, spatial heatmaps generated from fixation points and durations provide a comprehensive visualization of the spatial distribution of attention. Blink frequency and pupil diameter can also indicate attention levels and cognitive load to some extent. To explore the similarities and disparities in environmental attention when walking, jogging, and cycling, this study aims to simulate these three PPAs in a laboratory setting and collect eye movement metrics for comparative analysis.

The following hypotheses are proposed: (1) Since walking, jogging and cycling are all path-dependent physical activities, attention to the environment is common to them, and exercisers are likely to be drawn to common environmental features; (2) Owing to variations in intensity and speed among the three PPAs, attention distribution differs, making certain environmental features more critical for driving interest and maintaining attention during different activity states.

This research, based on the comparison of environmental attention levels and duration, seeks to provide deeper scientific evidence for the design and optimization of greenways, aiming at promoting diverse physical activities and ultimately enhancing their contribution to human health and wellbeing.

## 2 Literature review

### 2.1 Greenways and physical activity

In recent decades, greenways have been widely implemented in numerous countries and regions to promote active transportation and physical activity. Ireland’s “Greenways and Cycle Routes Ancillary Infrastructure Guidelines” emphasizes the imperative of developing and improving the planning as well as design aspects of greenways and cycle tracks to better cater to user needs (Blue Ridge Parkway, 2018). In the United States, the “Greenway Criteria and Design Guide” sets cycling path regulations to ensure the safety of cyclists along the greenway route (Department of Transport, and Tourism and Sport, 2018). The Blue Ridge Parkway in the US has successfully developed hiking, cycling, and horseback riding trails to support various recreational activities (East Coast Greenway, 2019). London’s Southeast “Green Chain” features diverse recreational paths, and health instructions are provided to guide exercisers in fitness walking.

Numerous studies have demonstrated that the introduction of greenways significantly increases the level of overall physical activity, especially moderate-to-vigorous physical activity (MVPA) (Coutts, 2008; Deng et al., 2023; Frank et al., 2021), decreases body mass index (BMI) (He et al., 2022), and enhances general physical health (Shafer et al., 2000). The more time spent on the greenway, the greater the benefits of physical activity (Deng et al., 2023). Accessibility plays an important moderating role, with the extent of physical activity improvement decreasing as the distance from the greenway increases (Frank et al., 2019). Appropriate design and timely renovation also matter. Zhang et al. (2022) found that improving sidewalks, bike paths, and greenway environments significantly impacts the potential for physical activity. Frank et al. (2019) discovered that the renovation of greenways doubled the likelihood of their use by nearby residents, while sedentary behavior decreased. However, positive effects are not always found. No improvement in physical activity levels was observed after the greenway construction in Northern Ireland (Hunter et al., 2021) and another low-income and disadvantaged community in Philadelphia (Auchincloss et al., 2019).

In general, most studies have only focused on overall physical activity levels and have not sufficiently explored specific activity programs.

### 2.2 Built environment and path-based physical activity

#### 2.2.1 Built environment and walking

Among the three PPAs, the impact of the built environment on walking has been studied the most. Overall, natural exposure (Xiao et al., 2024), perceived attractiveness (Ettema, 2016), path characteristics (Zhang and Mu, 2020) artificial facilities (Amati et al., 2018), and accessibility (Gong, 2023) all influence walking intensity and intention. Several studies have experimentally demonstrated that walking in green environments can regulate physiological responses (Briki and Majed, 2019), enhance attention levels (Xiao et al., 2024), and increase the willingness to walk (Kweon et al., 2021). Other studies have also investigated walkers’ preferences through survey questionnaires. The findings indicate that the physical conditions influencing walkers’ route choices encompass a variety of land uses as well as safe and comfortable sidewalk environments (Basu et al., 2023; Zhang and Mu, 2020). Additionally, objective data analysis has been utilized to evaluate walking accessibility. Gong (2023) employed Google Maps data to demonstrate that both walking time and street conditions significantly influence accessibility. Furthermore, highly attractive parks promote residents’ walking behavior. Beyond the spatial factors, temporal considerations have also been recognized as significant influences (Lindelow et al., 2014). In addition, the walkability index of the built environment is constantly being improved based on multi-scale features (Al Shammas et al., 2023; Alhajaj, 2023).

#### 2.2.2 Built environment and jogging

Among the three PPAs, the relationship between the built environment and jogging has been discussed the least. Studies have mainly focused on the influence of environmental types and characteristics on jogging intensity based on spatial trajectory observation and jogger preference via a questionnaire survey. Different types of environments provide varying levels of support for jogging. One study conducted a comparative analysis of various urban land-use types and identified that streets, residential areas, campuses, parks, and greenways possess significant potential to facilitate jogging (Tan et al., 2024). Another investigation indicated that joggers prefer parks over urban settings (Bodin and Hartig, 2003). Furthermore, a study demonstrated that green spaces and open areas enhance the appeal of jogging routes, particularly within parks and regions outside urban centers (Ettema, 2016). Environmental characteristics also play a crucial role in influencing jogging intensity, jogger satisfaction, and recovery. Street greenery and the distribution of blue-green spaces are positively associated with increased jogging intensity (Huang et al., 2023), while natural exposure is correlated with enhanced jogger satisfaction (Huang et al., 2022). Abundant vegetation and comfortable surfaces in greenway environments have been linked to enhanced recovery capacity (Deelen et al., 2019; Zhang et al., 2022).

#### 2.2.3 Built environment and cycling

Compared with walking and jogging, cycling is more common in the countryside, and large-scale natural landscapes are more stimulating for cyclists. Several studies have demonstrated that greenway environments exert a positive psychological influence on cyclists. Natural features such as vegetation and the sky elicit more favorable thoughts among cyclists (He et al., 2021). Perceptions of tree shapes and greenery while cycling have been shown to enhance mood and improve attention among university students (Zhang et al., 2023). Research utilizing cycling data and surveys is frequently employed to investigate cyclists’ route preferences. Cyclists generally favor routes characterized by well-developed infrastructure and scenic views (Desjardins et al., 2021; Willis et al., 2015). Female cyclists, in particular, tend to prefer straightforward and continuous paths (Rupi et al., 2023). Furthermore, Hu et al. (2023) proposed an evaluation framework for urban cycling environments based on shared bicycle trajectory data. In addition, various forms of greenways also affect the level of cycling activity (Wolff-Hughes et al., 2014).

Overall, few studies have compared walking, jogging, and cycling in the same framework. In addition, most studies take activity level and preference as analysis objects, and effective observation of exercisers’ perceptions of the environment is lacking.

### 2.3 Eye movement tracking in the urban green space study

Eye movement tracking is a technology that effectively observes human visual behaviors such as fixation, saccades, and scanning, providing insights into attention, cognition (Lu and Pesarakli, 2023), and emotions (Rak et al., 2020), and has been widely applied in urban green spaces in recent years. Eye-tracking technology has been employed to investigate visual behavior across various environments. Numerous studies have indicated that natural landscapes such as greenery and water bodies elicit varying levels of visual attention (Amati et al., 2018; Nordh et al., 2014; Zhou et al., 2023; Zhu et al., 2020). Conversely, other studies suggest that individuals tend to focus more on man-made elements such as roads (Gholami et al., 2021) or spend more time fixating on them (Amati et al., 2018). Furthermore, the color design of urban forest scenes can strongly attract visual attention (Chen et al., 2023). Eye-tracking analysis has also been integrated into investigations on aesthetic evaluation and restorative effects. Sun developed a comprehensive model for assessing the landscape quality of urban waterfront parks (Sun et al., 2018). Kang and Kim (2019) identified eye-tracking metrics that correlate with restorative qualities and suggested that using natural elements and considering complexity and openness can enhance the process of restoration.

## 3 Materials and methods

### 3.1 Visual material

Owing to its reliance on infrared light reflection, temperature fluctuations and strong light reflections can adversely impact the stability and sampling rate of the eye tracker. To mitigate these effects, a video was shot to simulate movement along a greenway route.

**FIGURE 1:**
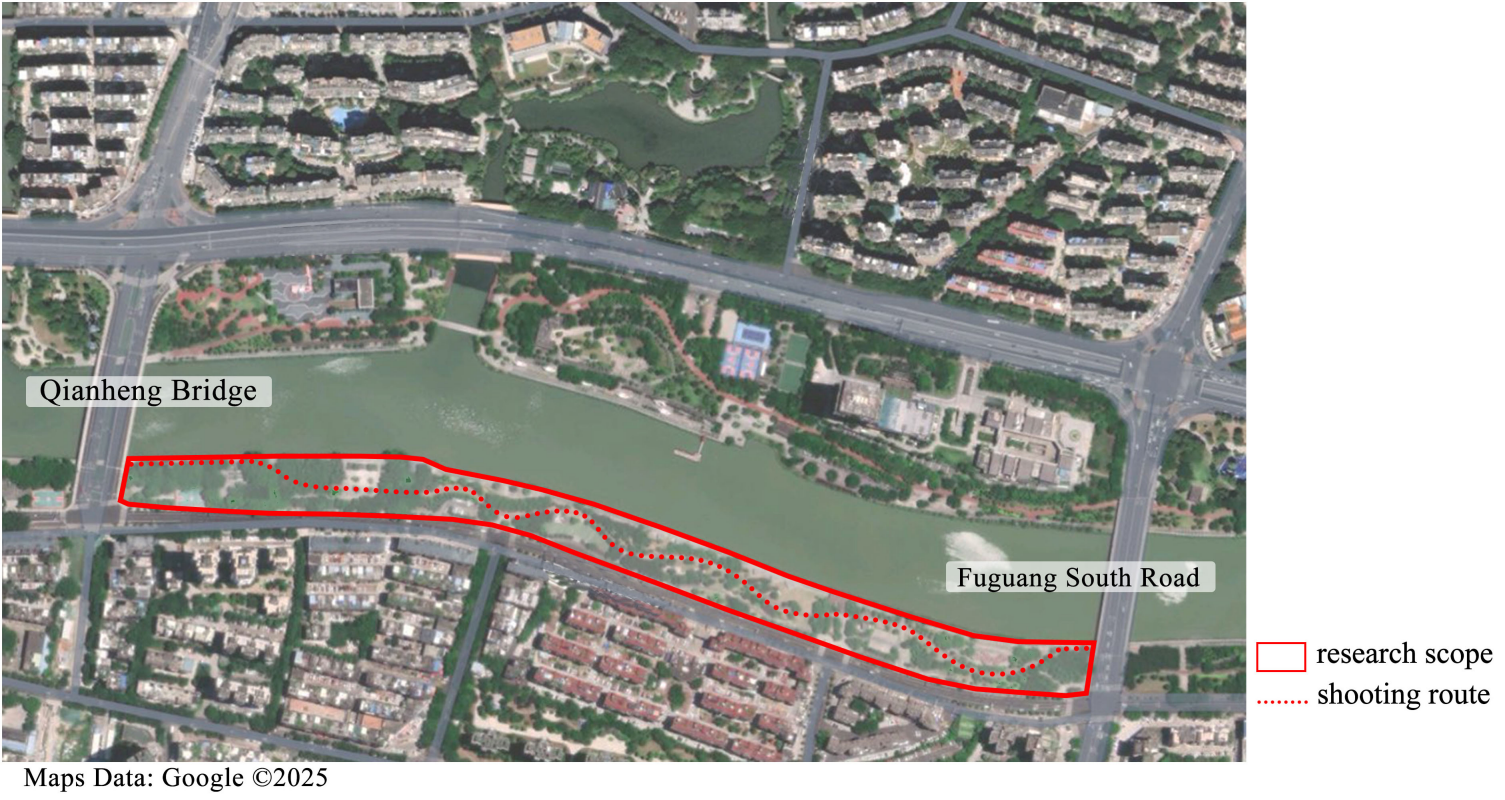
Shooting route (redrawn from Google Maps).

The video was shot in June 2023 during a calm, clear, and well-lit morning with minimal pedestrian traffic at Guangming Gang Park in Fuzhou, Fujian Province, China. The section selected for recording spanned from the southern part of Qianheng Bridge to Fuguang South Road (Figure 1), covering an approximate length of 1.2 km. To ensure video stability and accurately replicate the eye level during walking, jogging, and cycling, the GoPro9 action camera (23.6 MP, 2.7K resolution) was mounted on the photographer’s forehead using specialized gear. The entire video duration is 5 min and 20 s.

### 3.2 Experiment environment and devices

**FIGURE 2:**
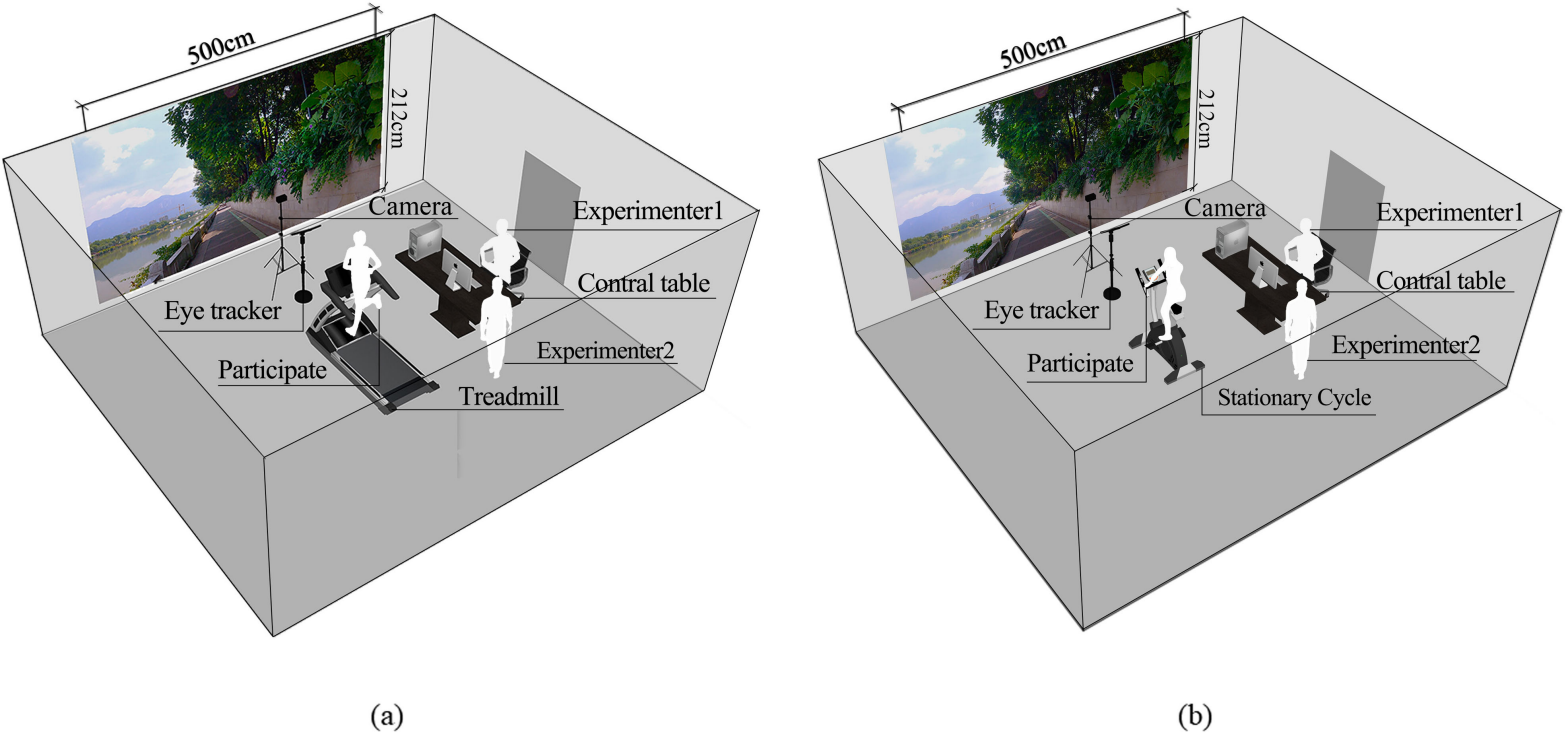
Layout of the experiment environment (source: author). **(a)** Walking and jogging sessions. **(b)** Cycling session. Experimenter 1 was responsible for monitoring the video and data; Experimenter 2 was responsible for setting up the exercise device and maintaining the safety of participants.

The experiment was conducted in a multimedia classroom equipped with a 500 cm × 212 cm (2500px × 1060px) LED screen, where the video content was displayed. Participants engaged in walking, jogging, and cycling on sport devices positioned at a distance of 1.7 meters from the screen (Figure 2). Walking and jogging were performed on a treadmill (model: SH-T9119P-H1, width: 660 mm, peak horsepower: 2.5 HP), while cycling was performed on a stationary cycle (model: B30, with 32 levels of electronically controlled resistance and a 9 kg flywheel).

A remote eye tracker (model: Tobii Pro Fusion, sampling rate: 250 Hz) was positioned directly in front of the participants. Since the eye tracker operates by detecting infrared light reflections to measure the position and movement of the eyes relative to the head, natural light was completely blocked during the experiment, and artificial lighting was employed instead.

### 3.3 Eye movement measurements

#### 3.3.1 Fixation of AOIs

**FIGURE 3:**
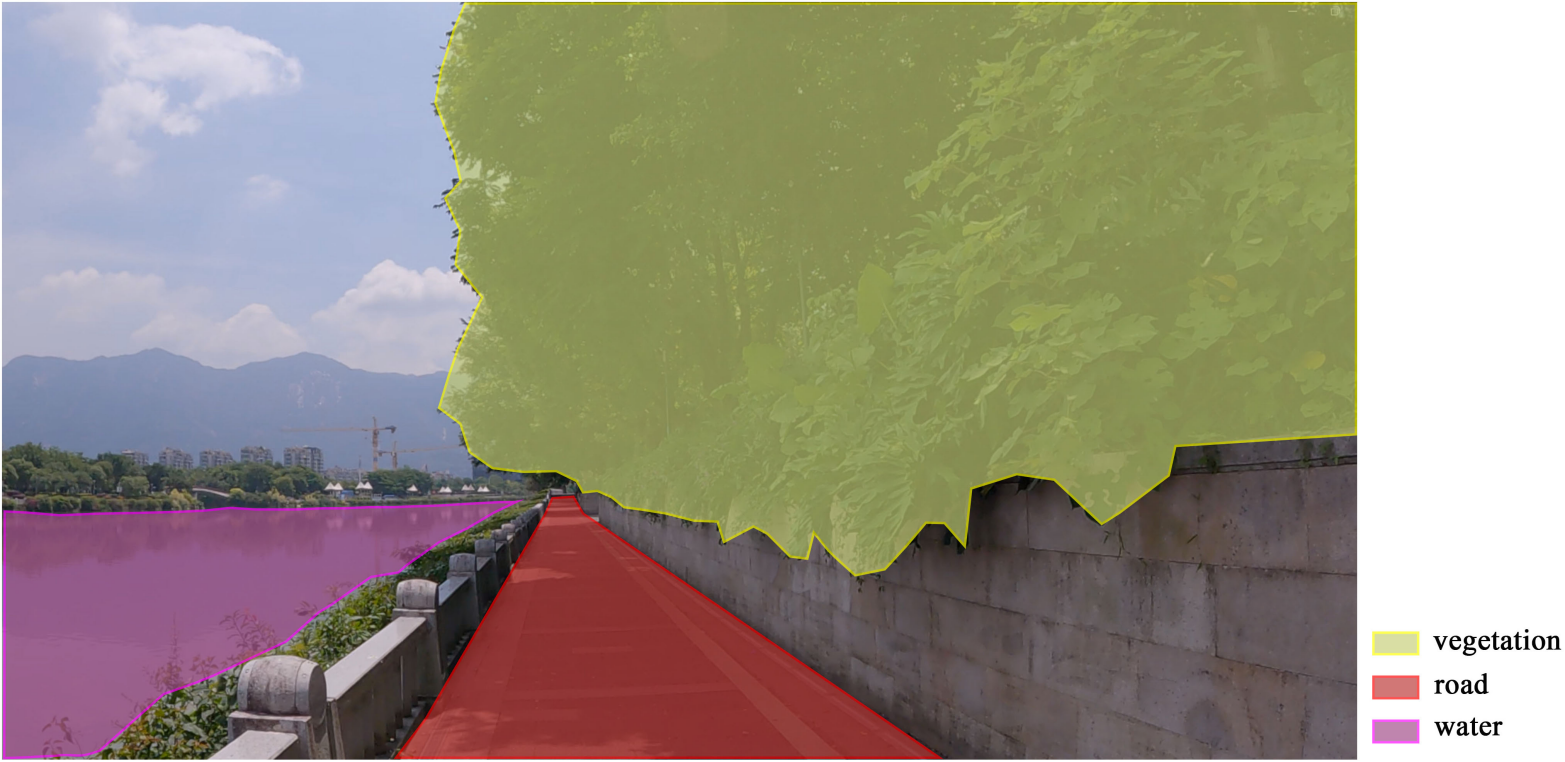
AOIs of an example frame (source: author).

Areas of interest (AOI) refer to specific portions of the visual material that are treated as independent analytical units. In this study, roads, water areas, and vegetation were defined as AOIs for fixation analysis. The researchers imported the video into the ErgoLAB 3.0 platform. Using the AOI tool within the eye-tracking module, a key frame was set every 10 s starting from the first frame to outline AOIs. The software automatically traced and generated AOIs for the intermediate frames. Finally, manual inspection and correction were performed for AOIs that exhibited displacement, overlap, or loss within the frames. The same video was used to delineate AOIs for the three types of PPA, ensuring complete synchronization in the scope and spatial positioning of the AOIs. The figure illustrates the AOI position distribution for a specific frame of the video (Figure 3). Due to the obstruction of water by vegetation or terrain in certain areas, it appears in non-continuous segments. In a 5-min and 20-s video, water is cumulatively visible for 2 min and 28 s, accounting for 46.3% of the total duration. In contrast, plant and road elements remain continuously visible throughout the entire video, with a visibility duration of 100%.

Fixation refers to the eye movement that stabilizes the retina on an object (Duchowski, 2003) and serves as the primary means of acquiring visual information, reflecting attention levels and depth of information processing. In this study, we assessed fixation behavior during movement using four metrics: duration of first fixation (DFF), total duration of fixation (TDF), number of fixations (NF), and average fixation duration (AFD). Since walking, jogging, and cycling speeds vary, video playback speed was adjusted according to the speed of the PPA, so the time of each PPA session was different. Therefore, a direct comparison of absolute metric values was inappropriate; instead, relative values were calculated (Lu and Pesarakli, 2023). For example, the relative DFF (rDFF) of a specific area of interest (AOI) refers to the ratio of that AOI’s DFF to the total DFF across all AOIs. The relative values of fixation metrics were calculated as shown in Equations 1–4:


$$r\,D\,F\,F_{i}=\frac{D\,F\,F_{i}}{\sum_{i=1}^{n}D\,F\,F_{i}}$$ (1)

$$\text{r}\,T\,D\,F_{i}=\frac{T\,D\,F_{i}}{\sum_{i=1}^{n}T\,D\,F_{i}}$$ (2)

$$r\,N\,F_{i}=\frac{N\,F_{i}}{\sum_{i=1}^{n}N\,F_{i}}$$ (3)

$$r\,A\,F\,D_{i}=\frac{A\,F\,D_{i}}{\sum_{i=1}^{n}A\,F\,D_{i}}$$ (4)

(1)–(4):*i* = 1,2,…, *n* (*n* = 3) represents the *i*-th area of interest (AOI).

#### 3.3.2 Blinks

The frequency of blinks serves as an indicator of attention concentration. Generally, a lower blinking frequency reflects a higher level of focused attention, whereas a higher blinking frequency suggests divided or distracted attention. Furthermore, sustained high levels of attention concentration are more likely to result in mental fatigue (Stern, 1994). In this study, the average blink rate (AB) per second was calculated and compared across walking, jogging, and cycling.

#### 3.3.3 Heatmap

The ErgoLAB eye-tracking analysis platform generates spatial patterns based on gaze position and duration, which are visually represented by specific shapes and color distributions. The size of these patterns reflects the range of attention distribution, while the color gradient indicates the duration of fixation in different regions. Warmer colors represent longer fixation durations, whereas cooler colors indicate shorter fixation durations.

### 3.4 Experiment procedure

The study aimed to compare the similarities and disparities in environmental visual attention across the three PPAs, rather than focusing on individual differences. A within-subject design was adopted, and eye-tracking data were repeatedly collected from each participant in all three PPAs. To minimize the effects of learning and fatigue, the order of the three PPAs was counterbalanced, generating six sequences: WJC, WCJ, JCW, JWC, CWJ, and CJW, where W indicates walking, J indicates jogging, and C indicates cycling. The experiment sequence was edited via the ErgoLAB platform.

**FIGURE 4:**
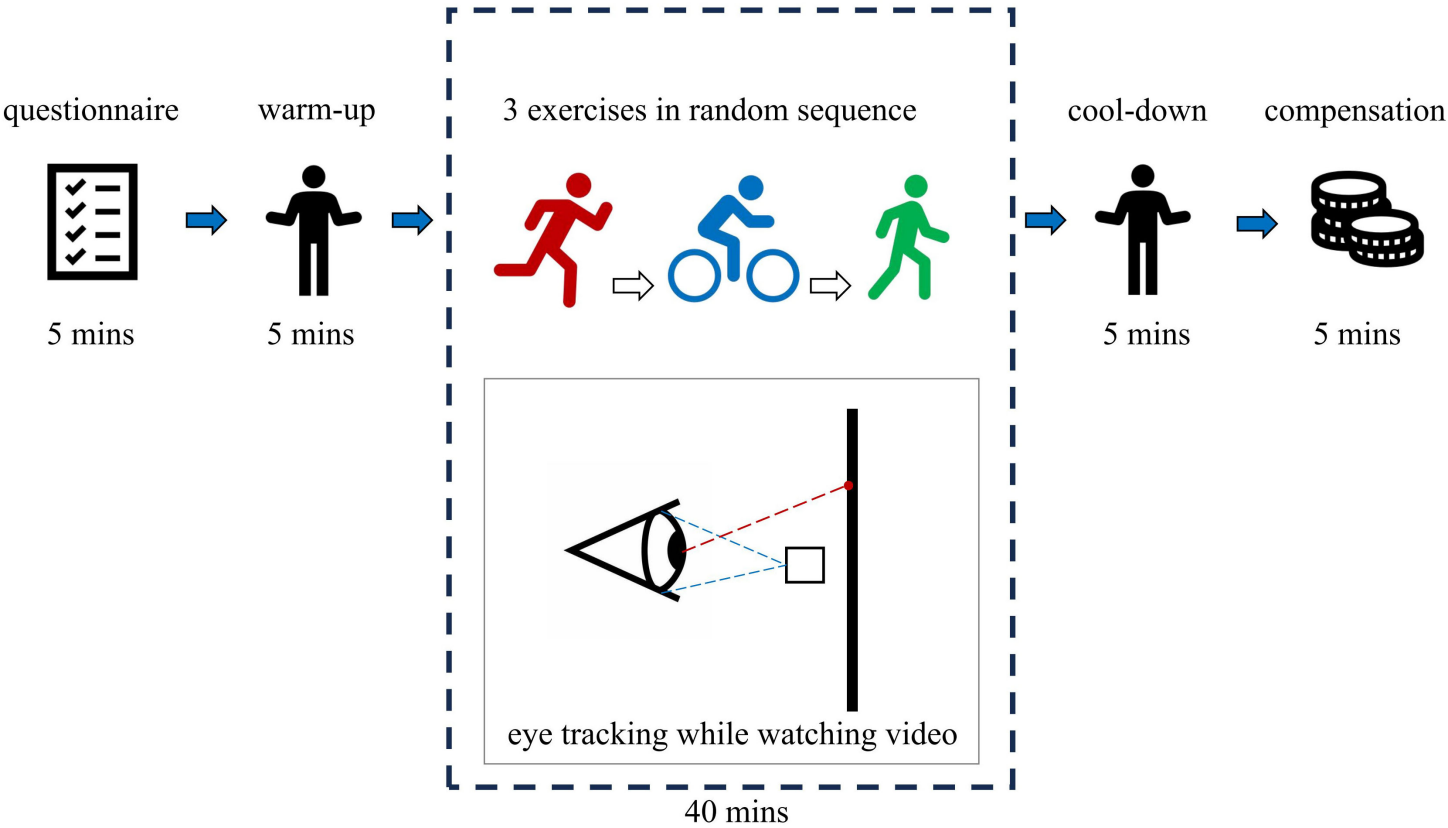
Flow chart of the experiment (source: author).

At the beginning of the experiment, each participant was randomly assigned one sequence of the six. After calibrating the eye tracker, the experimenter started the exercise device (treadmill or stationary cycle) and gradually increased its speed to reach the predetermined rate while allowing participants to warm up on the device. After they maintained a steady pace at the designated speed for 1 min, the video began to play. The three PPAs use the same video but vary in time and speed settings. During cycling, video playback lasted 5 min and 20 s at 13.5 km/h. Participants cycled on a stationary bike at a self-selected comfortable pace. The jogging video was played at 0.55× speed, lasting 9 min and 36 s, with both video and treadmill speeds set at 7.5 km/h. The walking video was played at 0.35× speed, lasting 15 min and 12 s, with both speeds maintained at 4.5 km/h. Before each PPA session, calibration of the eye tracker and a warm-up were conducted. Participants were instructed to maintain a steady pace while watching the video, without making significant head movements. Once all three PPAs were completed, participants received their compensation and left. The total experiment time for each participant was approximately 55 min (Figure 4).

### 3.5 Participants

A total of 30 university students, comprising 15 males and 15 females aged from 20 to 28 years old, volunteered to participate in the experiment. All participants had normal unaided or corrected vision, with no color blindness or other visual impairments, as well as normal eye size. Contact lenses and false eyelashes were prohibited during the experiment to ensure accurate measurements. To prevent exercise-related injuries, participants were allowed to stop the device and withdraw from the experiment at any time. Owing to the low valid sampling rate of the eye-tracking data from 4 participants, a final sample size of 26 was retained for analysis.

## 4 Results

The results of the normality test indicated that the collected eye-tracking data did not follow a normal distribution. Therefore, the Kruskal–Wallis non-parametric test was used for subsequent data analysis.

The analysis of attention throughout the entire exercise session used four metrics: AB, fixation density, gaze entropy and heatmap. For the analysis of attention within the AOIs, the metrics rDFF, rTDF, rNF, and rAFD were used.

### 4.1 Overall attention

#### 4.1.1 Average blink rate (AB)

**FIGURE 5:**
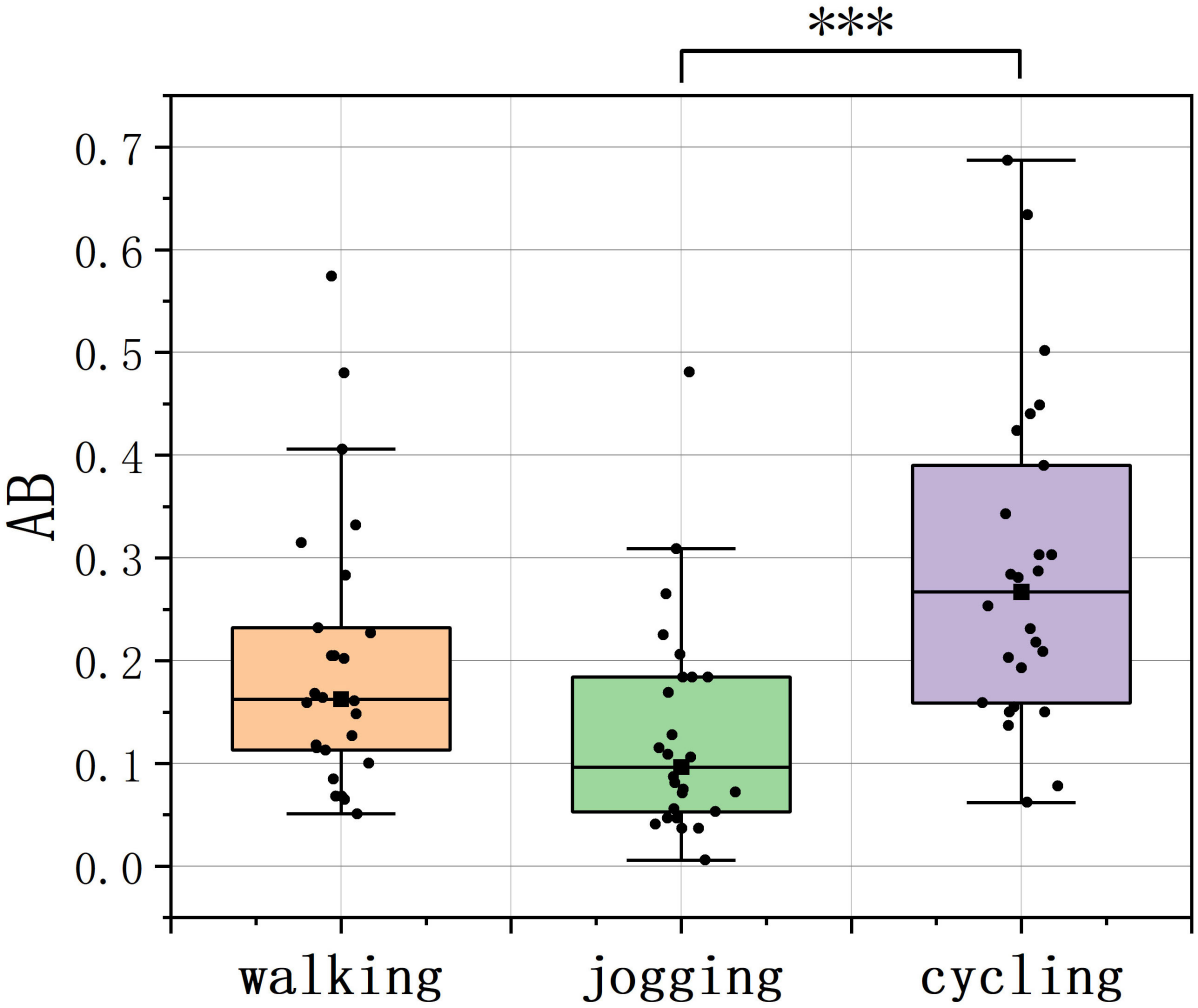
Data distribution of the AB across PPAs. ***p < 0.001. (source: author).

The Kruskal–Wallis test revealed a significant difference in AB among PPAs (H = 17.937, *p* < 0.001, ε^2^ = 0.693, Table 1). To identify which two of the three PPAs had differences in activities, pairwise comparisons were conducted with Bonferroni correction. The results indicated that during the entire exercise period, the AB during cycling was significantly higher than during jogging (Figure 5, *p* < 0.001), suggesting that individuals engaged in jogging exhibited a higher blinking frequency during physical activity.

**TABLE 1:** Kruskal–Wallis test results for eye movement metrics on different AOIs across PPAs.

Eye movement metrics	AOI	Walking *M* (P25, P75)	Jogging *M* (P25, P75)	Cycling *M* (P25, P75)	H	*p*-value	ε^2^
AB	\	0.16 (0.11, 0.24)	0.10 (0.05, 0.18)	0.27 (0.16, 0.40)	17.937	0.001	0.693
Gaze entropy	\	2.23 (2.08, 2.38)	1.86 (1.71, 1.95)	2.35 (2.16, 2.48)	34.551	0.001	0.434
rDFF	Road	0.28 (0.17, 0.37)	0.39 (0.22, 0.51)	0.27 (0.14, 0.46)	5.199	0.074	0.139
	Water	0.29 (0.14, 0.45)	0.30 (0.20, 0.44)	0.38 (0.18, 0.49)	0.762	0.683	0.000
	Vegetation	0.40 (0.26, 0.55)	0.29 (0.19, 0.37)	0.27(0.13, 0.47)	4.834	0.089	0.123
rTDF	Road	0.23 (0.12, 0.42)	0.39 (0.17, 0.57)	0.44 (0.23, 0.60)	4.837	0.089	0.123
	Water	0.03 (0.02, 0.04)	0.03 (0.02, 0.07)	0.04 (0.03, 0.06)	4.651	0.098	0.115
	Vegetation	0.74 (0.54, 0.83)	0.59 (0.39, 0.75)	0.51 (0.37, 0.68)	6.363	0.042	0.190
rNF	Road	0.23 (0.12, 0.39)	0.37 (0.20, 0.52)	0.41 (0.21, 0.54)	3.751	0.153	0.076
	Water	0.03 (0.02, 0.05)	0.03 (0.02, 0.05)	0.04 (0.03, 0.07)	6.578	0.037	0.199
	Vegetation	0.73 (0.56, 0.85)	0.60 (0.43, 0.77)	0.56 (0.41, 0.68)	5.607	0.061	0.157
rAFD	Road	0.35 (0.32, 0.37)	0.35 (0.33, 0.37)	0.37 (0.34, 0.40)	4.109	0.128	0.092
	Water	0.33 (0.30, 0.36)	0.33 (0.29, 0.37)	0.31 (0.26, 0.34)	3.862	0.145	0.081
	Vegetation	0.34 (0.31, 0.36)	0.31 (0.39, 0.33)	0.32 (0.31, 0.34)	3.618	0.164	0.070

The maximum of Fixation for each PPA is underlined.

#### 4.1.2 Fixation density, gaze entropy and heatmap

To further investigate differences in overall attention within the PPAs, fixation density and gaze entropy were analyzed. Fixation density was calculated based on the number of fixation across the entire screen for each PPA. Due to varying durations of the three movement types, data were standardized before comparison. The fixation density, from highest to lowest, were jogging (59.50), cycling (56.11), walking (54.73), indicating greater attention concentration in joggers.

Gaze entropy quantifies the spatial dispersion of fixation points to assess attention distribution. Lower entropy reflects more stable and focused attention, while higher entropy indicates more dispersed attention. The screen was divided into a 4 × 4 grid of equal sections, and gaze entropy was computed based on the distribution of fixation points across these grids. The calculation is as shown in Equation 5:


$$H=-\sum_{i=1}^{n}p_{i}\cdot \log_{2}p_{i}$$ (5)

* *i* = 1,2,…, *n* (*n* = 16) represents the *i*-th area

*pi represents the proportion of the number of fixations in the *i*-th region.

**FIGURE 6:**
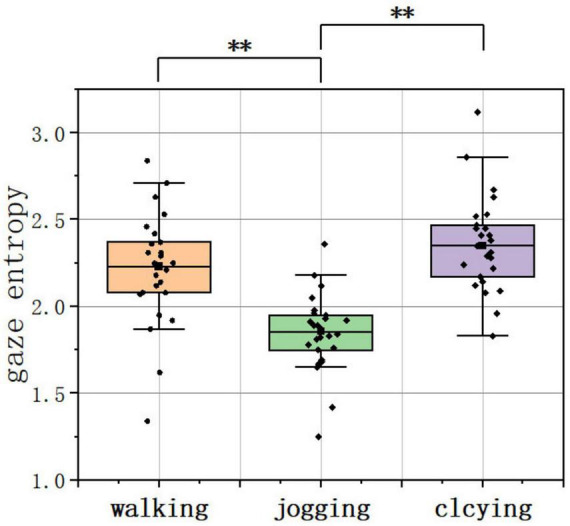
Data distribution of the gaze entropy across PPAs. **p < 0.01. (source: author).

The Kruskal–Wallis test on gaze entropy revealed significant differences among the three PPAs. Pairwise comparisons showed that joggers had significantly lower entropy than walkers and cyclists (*p* < 0.01, Figure 6), indicating greater attentional focus during jogging.

**FIGURE 7:**
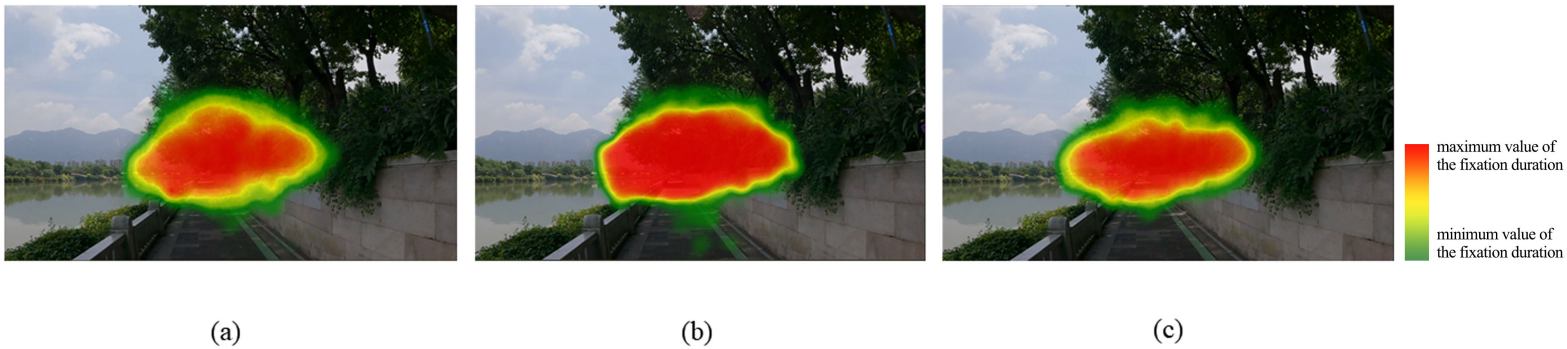
Heatmap based on the duration of fixation (source: author). **(a)** Walking; **(b)** jogging; **(c)** cycling.

The heatmap visually validates the above results. The heatmap for joggers displays the highest color saturation, indicating a more compact and concentrated distribution, with only minor dispersion observed along roads. Cyclists exhibit a more condensed and flattened heatmap pattern, whereas pedestrians demonstrate greater dispersion (Figure 7).

### 4.2 Fixation of AOIs

**FIGURE 8:**
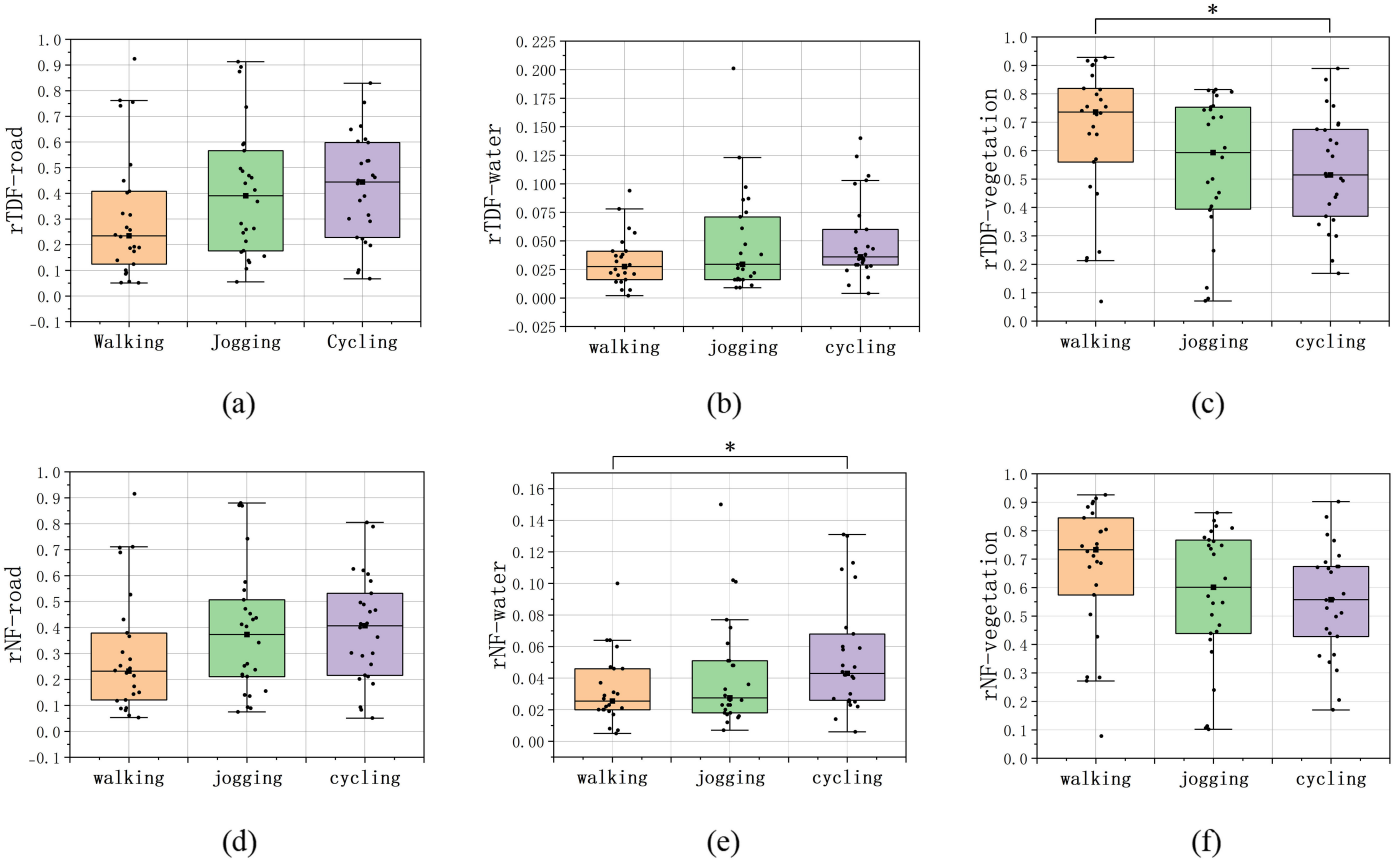
Data distribution of the rTDF and rNF on different AOIs across PPAs. *p < 0.05. (source: author). **(a)** rTDF on road AOI; **(b)** rTDF on water AOI; **(c)** rTDF on vegetation AOI; **(d)** rNF on road AOI; **(e)** rNF on water AOI; **(f)** rNF on vegetation AOI.

Despite differences in overall attention among PPAs, common patterns of attention allocation across regions of interest were observed. Median comparisons revealed that, across the three PPAs, vegetation received the longest rTDF and rNF (Table 1). The Kruskal–Wallis test indicated significant differences with a large effect size in rTDF among the three movement types (H = 6.363, *p* = 0.042, ε^2^ = 0.190) (Table 1). Pairwise comparisons showed that rTDF during walking was significantly higher than during cycling (*p* < 0.05, Table 1 and Figure 8).

Among the three PPAs, the water received the lowest rNF and rTDF (Table 1). Significant differences with large effect sizes were observed in rNF across the different PPAs (H = 6.578, *p* = 0.037, ε^2^ = 0.199, Table 1). Pairwise comparisons revealed that the rNF value during cycling was significantly higher than during walking (Figure 8).

Although no significant differences in fixation metrics on road, a comparison of the medians revealed that roads consistently showed the highest rAFD among the three PPAs. Moreover, joggers exhibited the maximum rDFF while running on roads (Table 1).

### 4.3 Exploratory analysis of gender differences

To investigate whether gender differences influenced the research results, an exploratory Kruskal–Wallis test was performed to examine potential gender differences in eye movement metrics across the three PPAs. The *p*-values were corrected using Bonferroni. For outcomes, *p* < 0.0013 was considered statistically significant. However, no significant differences were found. A partial report of descriptive statistics has been presented (Table 2). The complete tables of descriptive statistics and exploratory analysis results are available in the Supplementary material.

**TABLE 2:** Descriptive statistics of eye-tracking metrics among different genders across PPAs (Partial).

Eye movement metrics	Walking [*M* (P25, P75)]		Jogging [*M* (P25, P75)]		Cycling [*M* (P25, P75)]	
	Male	Female	Male	Female	Male	Female
rTDF Road	0.19 (0.11, 0.43)	0.27 (0.12, 0.46)	0.26 (0.16, 0.48)	0.44 (0.22, 0.67)	0.37 (0.22, 0.56)	0.47 (0.27, 0.63)
rNF Water	0.03 (0.02, 0.05)	0.03 (0.02, 0.04)	0.03 (0.02, 0.09)	0.03 (0.02, 0.05)	0.04 (0.02, 0.09)	0.05 (0.03, 0.07)
AB	0.14 (0.11, 0.22)	0.17 (0.11, 0.30)	0.07 (0.04, 0.14)	0.13 (0.07, 0.22)	0.23 (0.16, 0.42)	0.28 (0.18, 0.38)

## 5 Discussion

Based on the theoretical framework of ART, this article comprehensively explains the meaning of eye movement indicators need to integrate the context of movement and the task goals. Specifically, prolonged fixations on task-related content reflect cognitive investment in directed attention for spatial positioning, whereas prolonged fixations on naturally appealing features indicate interest driven by undirected attention. A detailed explanation will be provided in the discussion section based on the results of specific indicators.

### 5.1 Similarities and implications

In all three PPAs, vegetation consistently exhibited the highest rNF and the longest rTDF compared to the other two AOIs, multiple fixations may suggest that vegetation is visually appealing. Long time fixations are driven by interest and serve to sustain attention, rather than resulting from cognitive load. Previous studies have also confirmed the attractiveness of vegetation environments in linear open spaces visually assessed street pedestrians and found that trees were the most captivating element (Tabatabaie et al., 2023). Tilt (2007) using a photo questionnaire, identified a positive association between a higher canopy cover in communities and more frequent engagement in active walking experiences. Similarly, Van Holle et al. (2014) discovered that participants considered vegetation to be the most appealing environmental feature based on their intuitive reactions to photographs. In many cases, vegetation serves as the primary focal point for users of linear spaces such as greenways, streets, and scenic paths. Therefore, in future greenway design, vegetation planning could prioritize the following aspects. First, ensuring an adequate quantity of vegetation within the field of view is recommended. Research suggests that eye-level greenness has a greater impact on perception than absolute vegetation coverage (Huang et al., 2022). Even in areas where complete greening may not be feasible, the visibility of roadside vegetation should be maintained. Second, maintaining rich species diversity may significantly enhance people’s perception of environmental attractiveness (Deelen et al., 2019). One often overlooked but critical strategy is introducing variations in hierarchy, scale, and color to help avoid repetition and monotony, thereby continuously providing new visual stimuli engaged in physical exercise. This strategy may effectively alleviate mental fatigue during exercise.

Roads are an essential component of greenways. Compared to other AOIs, roads consistently elicited the longest rAFD among all three PPAs. Furthermore, joggers tend to elicit a lower AB when jogging on roads. This phenomenon does not indicate that exercisers are inherently interested in roads. Rather, it is driven by the necessity to perform navigation tasks to ensure safety, which requires cognitive investment in directed attention and is likely to impose a relatively high cognitive load. Similarly, Amati et al. (2018) discovered that park users allocate the highest proportion of their relative fixation time to roads, which they attributed to the task-oriented nature of way-finding navigation. And Gholami et al. (2021) noted that fixations during park visits were predominantly focused on roads and explained this pattern as a result of visual behavior associated with way-finding. Therefore, when designing greenways, it may be essential to implement differentiated treatments of road visual characteristics. The incorporation of colored asphalt pavements, painted guiding lines, and symbolic signage can enhance road visibility and facilitate the perception of road information, thereby reducing the elevated cognitive load associated with excessive directed attention. This is particularly important for elderly individuals with declining visual capabilities, as clearly recognizable roads can significantly improve the safety of their physical activities.

In this study, water exhibited the lowest rTDF, rNF, and rAFD compared to other AOIs. This may indeed reflect the relatively low attractiveness of the water. However, it could also be attributed to insufficient exposure time, which did not provide participants with sufficient opportunities for in-depth processing. Consistent with Nordh et al. (2014) eye-tracking study, the lower visual attention given to water compared to vegetation may be attributed to the lower frequency with which water appear as stimulus materials relative to other elements, but it still contributed to the perception of environmental restoration. Similarly, Zhou et al. (2023) observed that park visitors paid less attention to water compared to vegetation. Moreover, macro-scale studies have demonstrated that proximity to water (blue space) enhances environmental attractiveness as it is often perceived as dynamic and can energize exercisers (Deng et al., 2023; Pasanen et al., 2019; White et al., 2024). Additionally, open water bodies offer distant views and alleviate visual fatigue. However, both this study and previous researches indicate that water alone has a limited role in capturing attention. In design contexts, it might be advisable to consider integrating water with other landscape elements such as vegetation and architecture, and installing facilities like waterfront platforms and wading steps to explore the potential for enhancing hydrophilicity and landscape attractiveness.

### 5.2 Disparities and implications

The rDFF reflects immediate visual interest. Across the three PPAs, the AOI with the highest rDFF values differ, indicating variations in the factors that influence attention. In the case of walking, the vegetation elicited the highest rDFF. Given that vegetation are generally visually appealing, this elevated rDFF suggests interest-driven attentional behavior. With the longest rTDF, it further highlights the importance of both stimulating interest and sustaining attention. This may be attributed to the rich and intricate branching structures of vegetation and the slower pace of walking that allows for greater focus on these details. Previous studies focusing on walkers have similarly concluded that vegetation receives substantial attention in green spaces (Kweon et al., 2021; Xiao et al., 2024). In greenways specifically designed for slow-paced activities like walking, developing a design strategy that emphasizes vegetation in the creation of complex and engaging landscapes may prove to be an effective approach for enhancing the walking experience.

In terms of jogging, the road AOI inspired the longest rDFF. This may indicate that the road captures attention or that individuals allocate directed attention during spatial orientation. When considered alongside the relatively low AB value observed among joggers, the elevated rDFF value suggests a high degree of attention concentration and cognitive load. Among the three PPAs, jogging requires the highest intensity, demanding greater cognitive resources for maintaining balance. Data from fixation density, gaze entropy, and heatmap indicate that attention during jogging is highly concentrated and sustained. Therefore, when designing greenway running environments, careful consideration should be given to how the environment affects cognitive load. The landscape design could prioritize clear structure and organization in accordance with Kaplan’s preference theory principles such as legibility and coherence (Rachel Kaplan, 1989), which may help reduce the excessive cognitive load imposed by navigation tasks. At the same time, landscape variation should be introduced along the route, such as alternating open and enclosed spaces and incorporating diverse landscape nodes. This may shift directed attention to undirected attention, enhance exercisers’ interest, and promote psychological restoration.

Water was found to be the AOI eliciting the longest rDFF during cycling, which may suggest that water plays a significant role in capturing cyclists’ attention. Previous studies have also identified a spatial correlation between blue spaces and urban cycling activities (Jansen et al., 2017). Furthermore, gaze entropy and heatmap analyses reveal that cyclists’ fixation areas are broadly distributed and spatially dispersed. This pattern suggests that cyclists tend to focus around the vanishing point of perspective, likely due to their attention being directed toward more distant visual cues. Current greenway planning strategies supporting cycling often prioritize integration with large-scale blue spaces such as major urban rivers and lakes, as exemplified by popular projects like Donghu Greenway in Wuhan and Huangpu Riverside Greenway in Shanghai, China. Future research could further investigate cyclists’ attention to water in greater depth.

### 5.3 Limitations and prospects

The video-based indoor simulation experiment has limitations in environmental scanning, visual immersion, and attention allocation. First, regarding environmental scanning, in real-world settings, individuals rely on head movements and large-scale visual exploration to integrate information about their surroundings. Vestibular signals and proprioception regulating visual fixation (Schumann et al., 2008). However, in this study, participants were instructed to maintain stable head and neck positions throughout the experiment, they were limited to gather environmental information, leading to underutilization of peripheral vision, which in turn affected fixation. Papinutto et al. (2020) noted that the eyes compensate through more frequent and shorter fixations to cover a broader visual field when head movement were restricted, leading to an increase in fixation count and a decrease in average fixation duration.

Secondly, in terms of visual immersion, viewing videos on a fixed screen differs from experiencing a real environment due to limitations in field of view, optical flow, and depth perception. Zhao (2012) found that the field of view in simulated environments is narrower than that in real environments, which restricts the acquisition of visual information. Compared with real-world scenarios, watching a 2D display lacks depth cues. Moreover, accommodation-convergence conflict leads to inconsistent visual cues, resulting in inaccurate depth perception (Hartle and Wilcox, 2022). Additionally, the speed and variation of optical flow provide important depth cues for humans. In natural environments, optical flow aligns with human movement and perception, effectively regulating eye movement behavior. However, in simulation experiments, mismatches between optical flow speed and walking speed, as well as inconsistencies between stereoscopic parallax and optical flow, can cause conflicts in depth perception. These discrepancies may lead to eye-muscle fatigue and negatively affect blink frequency and fixation stability. Research has shown that the mismatch between video playback speed and walking speed in simulated environments results in decreased fixation duration compared to real-world conditions.

Finally, regarding attention allocation, there are differences in maintaining balance in attention allocation between the real environment and simulation experiments. The complexity of the real environment compels individuals to dynamically adjust their fixation strategies to maintain balance and ensure safety. For example, Foulsham et al. (2011) found that the real environment group spent more time fixating on the path to avoid obstacles, while the laboratory group fixated on the distance for a longer period. Drewes et al. (2021) discovered that body movements in the real environment involve more exploratory eye movements to adapt to environmental changes. Additionally, in the real environment, multisensory factors regulate the operation of peripheral attention. However, this study only provided visual stimuli, which reduced individuals’ active monitoring of the surrounding environment and may have led to an increase in the number and duration of fixations within the area.

Another limitation is the uneven presentation AOIs. Although we normalized the fixation duration metric to mitigate the influence of varying exposure times, the relatively low exposure frequency of the water AOI may still constrain the depth of cognitive processing. Manual annotation is susceptible to errors in defining AOI boundaries. Furthermore, when dealing with irregularly shaped objects such as plants, which cannot ensure absolute accuracy and may result in misclassification of fixation.

To address these limitations, future research could consider utilizing wearable eye-tracking devices in real-world environments to capture more ecologically valid data. Moreover, more precise tools should be sought for AOIs division and annotation to minimize errors.

## 6 Conclusion

This study compared the eye movement responses of participants during walking, jogging, and cycling while watching greenway videos, revealing the similarities and disparities in environmental attention among exercisers. In terms of similarities, vegetation, as the primary landscape element, consistently received the most attention across all three PPAs. Owing to their association with spatial orientation as a cognitive activity, roads necessitated relatively longer fixation each time. Regarding disparities, jogging exhibited the highest concentration of attention among the three PPAs, characterized by fewer blinks and greater visual reliance on the road. Cyclists’ attention was more driven by open water bodies and distant views, while walkers, moving at a slower pace, focused more on nearby vegetation landscapes. In terms of greenway design, clear and varied vegetation landscapes, distinct and noticeable roads, and open water surfaces supplying distant views can better match the attention modes of all three PPAs. These design features help sustain exercisers’ interest in the environment while reducing mental fatigue. This research provides scientific evidence on visual attention to support the design and optimization of greenways that accommodate multiple PPAs, enhancing their role in promoting physical activity and non-motorized transportation.

## Data Availability

The raw data supporting the conclusions of this article will be made available by the authors, without undue reservation.

## Supplementary material

Supplementary Table 1 The fixations on different AOIs across PPAs.

## References

[B1] Al Shammas T. Gullon P. Klein O. Escobar F. (2023). Development of a GIS-based walking route planner with integrated comfort walkability parameters. *Comp. Environ. Urban Syst.* 103:101981. 10.1016/j.compenvurbsys.2023.101981

[B2] Alhajaj N. (2023). Assessment of walkability of large parking lots on University campuses using walking infrastructure and user behavior as an assessment method for promoting sustainability. *Sustainability* 15:7203. 10.3390/su15097203

[B3] Amati M. Ghanbari Parmehr E. McCarthy C. Sita J. (2018). How eye-catching are natural features when walking through a park? Eye-tracking responses to videos of walks. *Urban For. Urban Green.* 31 67–78. 10.1016/j.ufug.2017.12.013

[B4] Auchincloss A. H. Michael Y. L. Kuder J. F. Shi J. Khan S. Ballester L. S. (2019). Changes in physical activity after building a greenway in a disadvantaged urban community: A natural experiment. *Prevent. Med. Rep.* 15:100941. 10.1016/j.pmedr.2019.100941 31338283 PMC6627031

[B5] Basu N. Oviedo-Trespalacios O. King M. (2023). What do pedestrians consider when choosing a route? The role of safety, security, and attractiveness perceptions and the built environment during day and night walking. *Cities* 143:104551. 10.1016/j.cities.2023.104551

[B6] Blue Ridge Parkway (2018). *Blue ridge parkway official website.* Asheville, NC: Blue Ridge Parkway.

[B7] Bodin M. Hartig T. (2003). Does the outdoor environment matter for psychological restoration gained through running? *Psychol. Sport Exerc.* 4 141–153. 10.1016/S1469-0292(01)00038-3

[B8] Briki W. Majed L. (2019). Adaptive effects of seeing green environment on psychophysiological parameters when walking or running. *Front. Psychol.* 10:252. 10.3389/fpsyg.2019.00252 30809177 PMC6379348

[B9] Chen Z. Huang Y. Shen Y. Fu W. Yao X. Huang J. (2023). How vegetation colorization design affects urban forest aesthetic preference and visual attention: An eye-tracking study. *Forests* 14:1491. 10.3390/f14071491

[B10] Coutts C. (2008). Greenway accessibility and physical-activity behavior. *Environ. Plann. B: Plann. Design* 35 552–563. 10.1068/b34

[B11] Deelen I. Janssen M. Vos S. Kamphuis C. Ettema D. (2019). Attractive running environments for all? A cross-sectional study on physical environmental characteristics and runners’ motives and attitudes, in relation to the experience of the running environment. *BMC Public Health* 19:366. 10.1186/s12889-019-6676-6 30940104 PMC6446270

[B12] Deng Y. Liang J. Chen Q. (2023). Greenway interventions effectively enhance physical activity levels-A systematic review with meta-analysis. *Front. Public Health* 11:1268502. 10.3389/fpubh.2023.1268502 38145067 PMC10745803

[B13] Department of Transport, and Tourism and Sport (2018). *Greenways and cycle routes ancillary infrastructure guidelines (34).* Ireland: Department of Transport, Tourism and Sport.

[B14] Desjardins E. Higgins C. D. Scott D. M. Apatu E. Paez A. (2021). Using environmental audits and photo-journeys to compare objective attributes and bicyclists’ perceptions of bicycle routes. *J. Transport Health* 22:101092. 10.1016/j.jth.2021.101092

[B15] Drewes J. Feder S. Einhäuser W. (2021). Gaze during locomotion in virtual reality and the real world. *Front. Neurosci.* 15:656913. 10.3389/fnins.2021.656913 34108857 PMC8180583

[B16] Duchowski A. T. (2003). *Eye tracking methodology: Theory and practice.* London: Springer.

[B17] East Coast Greenway (2019). *Greenway criteria and design guide (31).* Greenwich, CT: East Coast Greenway.

[B18] Ettema D. (2016). Runnable cities: How does the running environment influence perceived attractiveness, restorativeness and running frequency? *Environ. Behav.* 48 1127–1147. 10.1177/0013916515596364

[B19] Foulsham T. Walker E. Kingstone A. (2011). The where, what and when of gaze allocation in the lab and the natural environment. *Vis. Res.* 51 1920–1931. 10.1016/j.visres.2011.07.002 21784095

[B20] Frank L. D. Hong A. Ngo V. D. (2019). Causal evaluation of urban greenway retrofit: A longitudinal study on physical activity and sedentary behavior. *Prev. Med.* 123 109–116. 10.1016/j.ypmed.2019.01.011 30731094

[B21] Frank L. D. Hong A. Ngo V. D. (2021). Build it and they will cycle: Causal evidence from the downtown Vancouver Comox Greenway. *Transport Policy* 105 1–11. 10.1016/j.tranpol.2021.02.003

[B22] Gholami Y. Taghvaei S. H. Norouzian-Maleki S. Mansouri Sepehr R. (2021). Identifying the stimulus of visual perception based on Eye-tracking in Urban Parks: Case study of mellat park in tehran. *J. For. Res.* 26 91–100. 10.1080/13416979.2021.1876286

[B23] Gong F. Y. (2023). Modeling walking accessibility to urban parks using Google Maps crowdsourcing database in the high-density urban environments of Hong Kong. *Sci. Rep.* 13:20798. 10.1038/s41598-023-48340-w 38012216 PMC10682472

[B24] Han K. (2020). The effect of environmental factors and physical activity on emotions and attention while walking and jogging. *J. Leis. Res.* 52 619–641. 10.1080/00222216.2020.1788474

[B25] Hartle B. Wilcox L. M. (2022). Stereoscopic depth constancy for physical objects and their virtual counterparts. *J. Vis.* 22:9. 10.1167/jov.22.4.9 35315875 PMC8944385

[B26] He D. Lu Y. Xie B. Helbich M. (2022). How greenway exposure reduces body weight: A natural experiment in China. *Landscape Urban Plann.* 226:104502. 10.1016/j.landurbplan.2022.104502

[B27] He H. Li J. Lin X. Yu Y. (2021). Greenway cyclists’ visual perception and landscape imagery assessment. *Front. Psychol.* 12:541469. 10.3389/fpsyg.2021.541469 34093293 PMC8176027

[B28] Hu Y. Shao C. Wang S. Sun H. Sun P. Chu Z. (2023). Evaluating bicycling environments with trajectory data on shared bikes: A case study of Beijing. *J. Adv. Trans.* 2023:2560780. 10.1155/2023/2560780

[B29] Huang D. Jiang B. Yuan L. (2022). Analyzing the effects of nature exposure on perceived satisfaction with running routes: An activity path-based measure approach. *Urban For. Urban Green.* 68:127480. 10.1016/j.ufug.2022.127480

[B30] Huang D. Tian M. Yuan L. (2023). Sustainable design of running friendly streets: Environmental exposures predict runnability by volunteered geographic information and multilevel model approaches. *Sustainable Cities Soc.* 89:104336. 10.1016/j.scs.2022.104336

[B31] Hunter R. F. Adlakha D. Cardwell C. Cupples M. E. Donnelly M. Ellis G. (2021). Investigating the physical activity, health, wellbeing, social and environmental effects of a new urban greenway: A natural experiment (the PARC study). *Int. J. Behav. Nutr. Phys. Act.* 18:142. 10.1186/s12966-021-01213-9 34717650 PMC8557552

[B32] Jansen F. M. Ettema D. F. Kamphuis C. B. M. Pierik F. H. Dijst M. J. (2017). How do type and size of natural environments relate to physical activity behavior? *Health Place* 46 73–81. 10.1016/j.healthplace.2017.05.005 28511083

[B33] Kang Y. Kim E. J. (2019). Differences of restorative effects while viewing urban landscapes and green landscapes. *Sustainability* 11:2129. 10.3390/su11072129

[B34] Kweon B. Rosenblatt-Naderi J. Ellis C. D. Shin W. Danies B. H. (2021). The effects of pedestrian environments on walking behaviors and perception of pedestrian safety. *Sustainability* 13:8728. 10.3390/su13168728

[B35] Lindelow D. Svensson A. Sternudd C. Johansson M. (2014). What limits the pedestrian? Exploring perceptions of walking in the built environment and in the context of every-day life. *J. Trans. Health* 1 223–231. 10.1016/j.jth.2014.09.002

[B36] Lu Z. Pesarakli H. (2023). Seeing is believing: Using eye-tracking devices in environmental research. *Herd-Health Environ. Res. Design J.* 16 15–52. 10.1177/19375867221130806 36254371

[B37] Nordh H. Hagerhall C. M. Holmqvist K. (2014). *Tracking restorative components: Patterns in eye movements as a consequence of a restorative rating task.* Manchester: Landscape Research Group.

[B38] Papinutto M. Lao J. Lalanne D. Caldara R. (2020). Watchers do not follow the eye movements of Walkers. *Vis. Res.* 176 130–140. 10.1016/j.visres.2020.08.001 32882595

[B39] Pasanen T. P. White M. P. Wheeler B. W. Garrett J. K. Elliott L. R. (2019). Neighbourhood blue space, health and wellbeing: The mediating role of different types of physical. *Environ. Int.* 131:105016. 10.1016/j.envint.2019.105016 31352260

[B40] Rachel Kaplan S. K. (1989). *The experience of nature a psychological perspective.* Cambridge: Cambridge University Library.

[B41] Rak R. J. Majkowski A. Kołodziej M. Tarnowski P. Amparo A. Alonso-Betanzos A. (2020). Eye-Tracking analysis for emotion recognition. *Comp. Intell. Neurosci.* 2020:2909267. 10.1155/2020/2909267 32963512 PMC7492682

[B42] Rogerson M. Barton J. (2015). Effects of the visual exercise environments on cognitive directed attention, energy expenditure and perceived exertion. *Int. J. Environ. Res. Public Health* 12 7321–7336. 10.3390/ijerph120707321 26133125 PMC4515658

[B43] Rupi F. Freo M. Poliziani C. Postorino M. N. Schweizer J. (2023). Analysis of gender-specific bicycle route choices using revealed preference surveys based on GPS traces. *Trans. Policy* 133 1–14. 10.1016/j.tranpol.2023.01.001

[B44] Schumann F. Einhauser W. Vockeroth J. Bartl K. Schneider E. Konig P. (2008). Salient features in gaze-aligned recordings of human visual input during free exploration of natural environments. *J. Vis.* 8:12. 10.1167/8.14.12 19146313

[B45] Shafer C. S. Lee B. K. Turner S. (2000). A tale of three greenway trails: User perceptions related to quality of life. *Landscape Urban Plann.* 49 163–178. 10.1016/S0169-2046(00)00057-8

[B46] Stern J. (1994). Blink rate: A possible measure of fatigue. *Hum. Fact.* 36 285–297. 10.1177/001872089403600209 8070793

[B47] Sun L. Shao H. Li S. Huang X. Yang W. (2018). Integrated application of eye movement analysis and beauty estimation in the visual landscape quality estimation of urban waterfront park. *Int. J. Pattern Recogn. Art. Intell.* 32:1856010. 10.1142/S0218001418560104

[B48] Tabatabaie S. Litt J. S. Muller B. H. F. (2023). Sidewalks, trees and shade matter: A visual landscape assessment approach to understanding people’s preferences for walking. *Urban For. Urban Green.* 84:127931. 10.1016/j.ufug.2023.127931

[B49] Tan L. Jiang J. Guo M. Zhong Y. (2024). Comparing differences in jogging support across various land use types in urban built-up areas using user-recommended routes. *Buildings* 14:851. 10.3390/buildings14030851

[B50] Tilt J. H. (2007). *Neighborhood vegetation and preferences: Exploring walking behaviors in urban and suburban environments*. Ann Arbor, MI: ProQuest Information and Learning Company.

[B51] Van Holle V. Van Cauwenberg J. Deforche B. Goubert L. Maes L. Nasar J. (2014). Environmental invitingness for transport-related cycling in middle-aged adults: A proof of concept study using photographs. *Trans. Res. Part A Policy Pract.* 69 432–446. 10.1016/j.tra.2014.09.009

[B52] White R. L. NicoleTaylor, Dean D. Cotton W. Peralta L. Young C. (2024). A systematic observation of moderate-to-vigorous physical activity levels in Australian natural blue space locations. *Health Promot. Int.* 39:daae101. 10.1093/heapro/daae101 39180351 PMC11344178

[B53] Willis D. P. Manaugh K. El-Geneidy A. (2015). Cycling under influence: Summarizing the influence of perceptions, attitudes, habits, and social environments on cycling for transportation. *Int. J. Sustainable Trans.* 9 565–579. 10.1080/15568318.2013.827285

[B54] Wolff-Hughes D. L. Fitzhugh E. C. Bassett D. R. Cherry C. R. (2014). Greenway siting and design: Relationships with physical activity behaviors and user characteristics. *J. Phys. Act. Health* 11 1105–1110. 10.1123/jpah.2012-0444 23962916

[B55] Xiao X. Li X. Zhou X. Kang J. Luo J. Yin L. (2024). Modulatory effects of the landscape sequences on pedestrians emotional states using EEG. *Front. Arch. Res.* 13 1327–1341. 10.1016/j.foar.2024.05.002

[B56] Zhang D. Jin X. Wang L. Jin Y. (2023). Form and color visual perception in green exercise: Positive effects on attention, mood, and self-esteem. *J. Environ. Psychol.* 88:102028. 10.1016/j.jenvp.2023.102028

[B57] Zhang X. Mu L. (2020). The perceived importance and objective measurement of walkability in the built environment rating. *Environ. Plann. B: Urban Anal. City Sci.* 47 1655–1671. 10.1177/2399808319832305

[B58] Zhang Y. Koene M. Reijneveld S. A. Tuinstra J. Broekhuis M. van der Spek S. (2022). The impact of interventions in the built environment on physical activity levels: A systematic umbrella review. *Int. J. Behav. Nutrit. Phys. Act.* 19:156. 10.1186/s12966-022-01399-6 36550583 PMC9773501

[B59] Zhao M. (2012). *Investigation of eye movements during walking in real and simulated environments.* PhD thesis, University of York: England.

[B60] Zhou X. Cen Q. Qiu H. (2023). Effects of urban waterfront park landscape elements on visual behavior and public preference: Evidence from eye-tracking experiments. *Urban For. Urban Green.* 82:127889. 10.1016/j.ufug.2023.127889

[B61] Zhu X. Zhang Y. Zhao W. (2020). Differences in environmental information acquisition from urban green—a case study of qunli national wetland park in Harbin, China. *Sustainability* 12:8128. 10.3390/su12198128

