# Correction: Does environmental attention differ during walking, jogging, and cycling in greenways? Evidence from eye movement responses to videos

**DOI:** 10.3389/fpsyg.2025.1727294

**Published:** 2025-11-14

**Authors:** Frontiers Production Office

**Affiliations:** Frontiers Media SA, Lausanne, Switzerland

**Keywords:** greenway, physical activity, health promotion, visual attention, eye-movement tracking

**Equation 5** in section: **Overall attention**, subsection: *Fixation density, gaze entropy and heatmap*, 2^nd^ Paragraph was erroneously given as


$$\begin{array}{l} H=-\overset{n}{\underset{i-1}{\sum }}pi\log_{2}pi\\ H=-\overset{n}{\underset{i=1}{\sum }}p_{i}.\log_{2}p_{i} \end{array}$$

The original version of this article has been updated.

There was a mistake in Tables 1 and 2 as published. The Table header was not integrated correctly. The previous version of the Tables 1 and 2 appear below.

**Table 1:** Kruskal–Wallis test results for eye movement metrics on different AOIs across PPAs.

		**Median (P25,P75)**					
**Eye movement metrics**	**PPA AOI**	**Walking**	**Jogging**	**Cycling**	***H***	***p*** **-value**	ε^2^
AB	\	0.16 (0.11, 0.24)	0.10 (0.05, 0.18)	0.27 (0.16, 0.40)	17.937	0.001	0.693
Gazeentropy	\	2.23 (2.08, 2.38)	1.86 (1.71, 1.95)	2.35 (2.16, 2.48)	34.551	0.001	0.434
rDFF	Road	0.28 (0.17, 0.37)	0.39 (0.22, 0.51)	0.27 (0.14, 0.46)	5.199	0.074	0.139
	Water	0.29 (0.14, 0.45)	0.30 (0.20, 0.44)	0.38 (0.18, 0.49)	0.762	0.683	0.000
	Vegetation	0.40 (0.26, 0.55)	0.29 (0.19, 0.37)	0.27 (0.13, 0.47)	4.834	0.089	0.123
rTDF	Road	0.23 (0.12, 0.42)	0.39 (0.17, 0.57)	0.44 (0.23, 0.60)	4.837	0.089	0.123
	Water	0.03 (0.02, 0.04)	0.03 (0.02, 0.07)	0.04 (0.03, 0.06)	4.651	0.098	0.115
	Vegetation	0.74 (0.54, 0.83)	0.59 (0.39, 0.75)	0.51 (0.37, 0.68)	6.363	0.042	0.190
rNF	Road	0.23 (0.12, 0.39)	0.37 (0.20, 0.52)	0.41 (0.21, 0.54)	3.751	0.153	0.076
	Water	0.03 (0.02, 0.05)	0.03 (0.02, 0.05)	0.04 (0.03, 0.07)	6.578	0.037	0.199
	Vegetation	0.73 (0.56, 0.85)	0.60 (0.43, 0.77)	0.56 (0.41, 0.68)	5.607	0.061	0.157
rAFD	Road	0.35 (0.32, 0.37)	0.35 (0.33, 0.37)	0.37 (0.34, 0.40)	4.109	0.128	0.092
	Water	0.33 (0.30, 0.36)	0.33 (0.29, 0.37)	0.31 (0.26, 0.34)	3.862	0.145	0.081
	Vegetation	0.34 (0.31, 0.36)	0.31 (0.39, 0.33)	0.32 (0.31, 0.34)	3.618	0.164	0.070

The maximum of fixation for each PPA is underlined.

**Table 2:** Descriptive statistics of eye-tracking metrics among different genders across PPAs (Partial).

**Eye movement metrics**		**Median (P25,P75)**					
	**PPAAOI**	**Walking**		**Jogging**		**Cycling**	
		**Male**	**Female**	**Male**	**Female**	**Male**	**Female**
rTDF	Road	0.19 (0.11, 0.43)	0.27 (0.12, 0.46)	0.26 (0.16, 0.48)	0.44 (0.22, 0.67)	0.37 (0.22, 0.56)	0.47 (0.27, 0.63)
rNF	Water	0.03 (0.02, 0.05)	0.03 (0.02, 0.04)	0.03 (0.02, 0.09)	0.03 (0.02, 0.05)	0.04 (0.02, 0.09)	0.05 (0.03, 0.07)
AB	\	0.14 (0.11, 0.22)	0.17 (0.11, 0.30)	0.07 (0.04, 0.14)	0.13 (0.07, 0.22)	0.23 (0.16, 0.42)	0.28 (0.18, 0.38)

The corrected Tables 1 and 2 appear below.

**Table 1:** Kruskal–Wallis test results for eye movement metrics on different AOIs across PPAs.

**Eye movement metrics**	**AOI**	**Walking *M* (P25, P75)**	**Jogging *M* (P25, P75)**	**Cycling *M* (P25, P75)**	***H***	***p*-value**	**ε^2^**
AB	\	0.16 (0.11, 0.24)	0.10 (0.05, 0.18)	0.27 (0.16, 0.40)	17.937	0.001	0.693
Gaze entropy	\	2.23 (2.08, 2.38)	1.86 (1.71, 1.95)	2.35 (2.16, 2.48)	34.551	0.001	0.434
rDFF	Road	0.28 (0.17, 0.37)	0.39 (0.22, 0.51)	0.27 (0.14, 0.46)	5.199	0.074	0.139
	Water	0.29 (0.14, 0.45)	0.30 (0.20, 0.44)	0.38 (0.18, 0.49)	0.762	0.683	0.000
	Vegetation	0.40 (0.26, 0.55)	0.29 (0.19, 0.37)	0.27 (0.13, 0.47)	4.834	0.089	0.123
rTDF	Road	0.23 (0.12, 0.42)	0.39 (0.17, 0.57)	0.44 (0.23, 0.60)	4.837	0.089	0.123
	Water	0.03 (0.02, 0.04)	0.03 (0.02, 0.07)	0.04 (0.03, 0.06)	4.651	0.098	0.115
	Vegetation	0.74 (0.54, 0.83)	0.59 (0.39, 0.75)	0.51 (0.37, 0.68)	6.363	0.042	0.190
rNF	Road	0.23 (0.12, 0.39)	0.37 (0.20, 0.52)	0.41 (0.21, 0.54)	3.751	0.153	0.076
	Water	0.03 (0.02, 0.05)	0.03 (0.02, 0.05)	0.04 (0.03, 0.07)	6.578	0.037	0.199
	Vegetation	0.73 (0.56, 0.85)	0.60 (0.43, 0.77)	0.56 (0.41, 0.68)	5.607	0.061	0.157
rAFD	Road	0.35 (0.32, 0.37)	0.35 (0.33, 0.37)	0.37 (0.34, 0.40)	4.109	0.128	0.092
	Water	0.33 (0.30, 0.36)	0.33 (0.29, 0.37)	0.31 (0.26, 0.34)	3.862	0.145	0.081
	Vegetation	0.34 (0.31, 0.36)	0.31 (0.39, 0.33)	0.32 (0.31, 0.34)	3.618	0.164	0.070

The maximum of fixation for each PPA is underlined.

**Table 2:** Descriptive statistics of eye-tracking metrics among different genders across PPAs (Partial).

**Eye movement metrics**	**Walking [*****M*** **(P25, P75)]**		**Jogging [*****M*** **(P25, P75)]**		**Cycling [*****M*** **(P25, P75)]**	
	**Male**	**Female**	**Male**	**Female**	**Male**	**Female**
rTDF-Road	0.19 (0.11, 0.43)	0.27 (0.12, 0.46)	0.26 (0.16, 0.48)	0.44 (0.22, 0.67)	0.37 (0.22, 0.56)	0.47 (0.27, 0.63)
rNF-Water	0.03 (0.02, 0.05)	0.03 (0.02, 0.04)	0.03 (0.02, 0.09)	0.03 (0.02, 0.05)	0.04 (0.02, 0.09)	0.05 (0.03, 0.07)
AB	0.14 (0.11, 0.22)	0.17 (0.11, 0.30)	0.07 (0.04, 0.14)	0.13 (0.07, 0.22)	0.23 (0.16, 0.42)	0.28 (0.18, 0.38)

